# Chitosan-capped enzyme-responsive hollow mesoporous silica nanoplatforms for colon-specific drug delivery

**DOI:** 10.1186/s11671-020-03351-8

**Published:** 2020-06-01

**Authors:** Defu Cai, Cuiyan Han, Chang Liu, Xiaoxing Ma, Jiayi Qian, Jianwen Zhou, Yue Li, Yiming Sun, Changting Zhang, Wenquan Zhu

**Affiliations:** 1 Institute of Medicine and Drug Research, Qiqihar Medical University, Qiqihar, 161006 China; 2 College of Pharmacy, Qiqihar Medical University, Qiqihar, 161006 China; 3 Department of Andrology, HeiLongJiang Hospital of Traditional Chinese Medicine, Harbin, 150036 China

**Keywords:** Hollow mesoporous silica spheres, Colon specific drug delivery, Enzyme-responsive, Doxorubicin

## Abstract

An enzyme-responsive colon-specific delivery system was developed based on hollow mesoporous silica spheres (HMSS) to which biodegradable chitosan (CS) was attached via cleavable azo bonds (HMSS–N=N–CS). Doxorubicin (DOX) was encapsulated in a noncrystalline state in the hollow cavity and mesopores of HMSS with the high loading amount of 35.2%. In vitro drug release proved that HMSS–N=N–CS/DOX performed enzyme-responsive drug release. The grafted CS could increase the biocompatibility and stability and reduce the protein adsorption on HMSS. Gastrointestinal mucosa irritation and cell cytotoxicity results indicated the good biocompatibility of HMSS and HMSS–N=N–CS. Cellular uptake results indicated that the uptake of DOX was obviously increased after HMSS–N=N–CS/DOX was preincubated with a colonic enzyme mixture. HMSS–N=N–CS/DOX incubated with colon enzymes showed increased cytotoxicity, and its IC_50_ value was three times lower than that of HMSS–N=N–CS/DOX group without colon enzymes. The present work lays the foundation for subsequent research on mesoporous carriers for oral colon-specific drug delivery.

## Introduction

Recently, stimuli-responsive drug delivery systems (DDSs) have attracted extensive attention for the efficient loading and selective release of drugs in targeted diseased tissues [1]. The designed stimuli-responsive systems can be delivered to diseased sites and realize on-demand drug release to improve therapeutic effects and hinder premature leakage-induced side effects. All internal and external stimuli, such as redox potential [2], pH [1], enzymes [3], and temperature and light [4, 5], have been used to design stimuli-responsive DDSs. Among these stimuli, enzymes, as internal stimuli, have gained wide attention due to their distinct concentrations in different tissues [6].

Over the past two decades, mesoporous silica spheres (MSSs) with mesopores ranging from 2–50 nm in size have been established as stimuli-responsive drug carriers [7, 8] since MSSs have a remarkably large pore volume and high surface area for high drug loading capacity, well-organized pore structure, an easily functionalized surface, and good biocompatibility [9]. Furthermore, hollow mesoporous silica sphere (HMSS) nanoparticles with a mesoporous shell structure and a hollow cavity are superior to conventional MSSs because the hollow structure can efficiently hold more drugs with a higher storage capacity than MSSs carriers [10, 11]. All kinds of stimuli-responsive nanocarriers based on MSSs were developed to host drug molecules using various gatekeepers, such as polymers [12], inorganic nanoparticles [13], dendrimers, biomacromolecules [14], macrocyclic compounds peptides [15], and lipids [16]. Although numerous DDSs based on MSSs with functional capping can realize release in response to various external or internal stimuli, few of them have been used in colon-specific targeted drug delivery.

It is well known that oral drug delivery is the favorite and a simple way of administering drugs. Colon-specific targeted drug delivery is very fascinating for the treatment of colonic diseases, including Crohn’s disease, colorectal cancer, and ulcerative colitis. However, colon-specific drug delivery might encounter several troubles, including there being less water content and relatively less surface for oral adsorption there than at other sites in the gastrointestinal (GI) tract [17–19]. Furthermore, oral DDSs also meet a strong acidic environment in the stomach, which might accelerate the degradation of loaded drugs in the GI tract, thus removing the ability to realize colonic targeted delivery [19]. For this reason, several pH-dependent DDSs have been designed to realize pH-triggered drug release at the nearly neutral pH values (6–7) of the GI tract, resisting the highly acidic conditions in the stomach region [20–23]. Only a slight difference in acidity between the intestinal (pH 6.8) and colonic (pH 7.4) regions exists; hence, such pH-responsive DDSs have difficulty realizing colon-specific release.

Chitosan (CS), a cationic and biodegradable naturally present polysaccharide, is constituted by β-(1-4)-linked glucosamine and N-acetyl-D-glucosamine units [24]. CS has received great attention in biological medicine owing to its fascinating properties, including biodegradability, biocompatibility, mucoadhesivity, and antibacterial activity [25–29]. Compared with polymers, polyelectrolytes, and supramolecules synthesized via complicated processes, CS is relatively cheap and readily available by the exhaustive deacetylation of chitin [30–32]. In addition, it has been reported that CS can open tight junctions between cells, thus increasing drug absorption [33]. Therefore, the polymer CS was selected as a capping agent owing to its good biocompatibility and appropriate size to cover the mesopores of HMSS to block drug release.

**Scheme 1:**
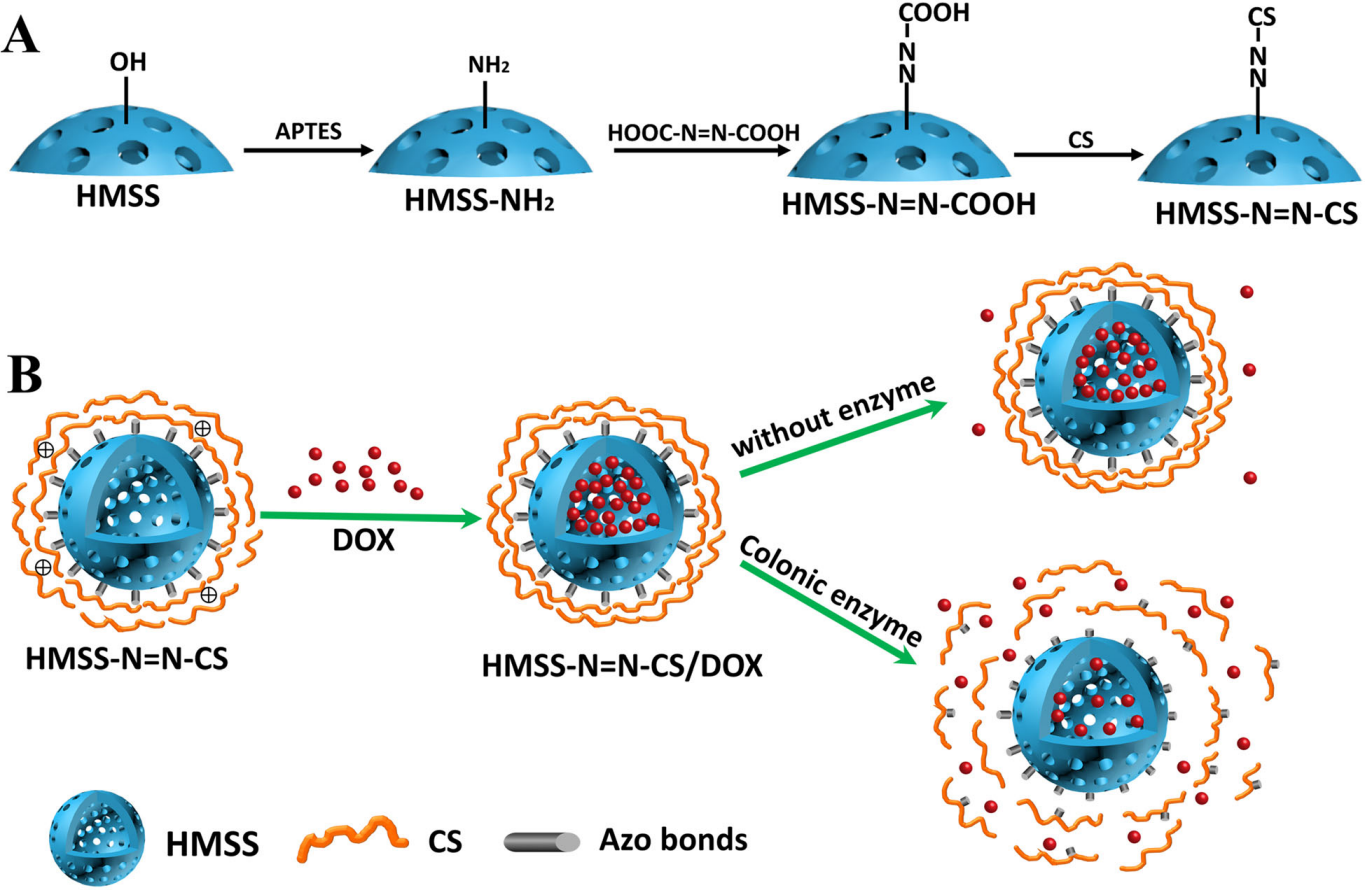
Schematic illustration of **a** preparation process of HMSS–N=N–CS and **b** the drug loading and enzyme-responsive release of HMSS–N=N–CS/DOX in response to colon enzyme

In our work, a colon-specific enzyme-responsive DDS based on an HMSS material (HMSS–N=N–CS) was designed for the first time as displayed in Scheme 1. In this system, the HMSS carriers were prepared via a selective etching strategy. The polymer CS was attached to the surface of HMSS by azo bonds to act as a gatekeeper to block the openings of HMSS. The azo bonds between HMSS and CS can be cleaved by enzymes in colon sites [34, 35], resulting in the separation of CS from the openings of HMSS. DOX was used as the model drug to be embedded into the cavity of HMSS, and in vitro drug release experiments were conducted to evaluate the enzyme-responsive release in the presence of colonic enzymes. Confocal laser scanning microscopy (CLSM) and flow cytometry (FCM) were used to investigate cellular uptake by Caco-2 cells. Finally, the cytotoxicity of HMSS–N=N–CS/DOX towards Caco-2 cells was measured.

## Materials and Methods

### Materials

Tetraethoxysilane (TEOS); N-(3-dimethylaminopropyl)-N-ethylcarbodiimide hydrochloride (EDC); chitosan (DAC ≥ 95%); cetyltrimethylammonium bromide (CTAB); 3-aminopropyltriethoxysilane (APTES); 3-(4,5-dimethylthiazol-2-yl)-2,5-diphenyl tetrazolium bromide (MTT); azobenzene-3,3′-dicarboxylic acid; potassium bromide (spectral purity, ≥ 99.5%); N-hydroxysuccinimide (NHS) and DOX were purchased from Aladdin Chemical Inc. (Shanghai, China). Azobenzene-3,3′-dicarboxylic acid was supplied by Inno-chem Technology Co. Ltd. (Beijing, China). Cell culture media DMEM, penicillin-streptomycin, and fetal bovine serum (FBS) were supplied by GIBCO, Invitrogen Co. (Carlsbad, USA). All analytical reagents were not further purified before use.

### Preparation of HMSS–N=N–CS

#### Preparation of HMSS–NH_2_

The HMSS nanoparticles were prepared based on the published work using a selective etching method [36]. The solid silica spheres were firstly synthesized by a modified Stober method. Briefly, 6 mL TEOS was poured into the mixture of 10 mL deionized water, 74 mL ethanol, and 3 mL concentrated NH_3_·H_2_O. Subsequently, the mixture was stirred for 60 min to obtain colloidal silica suspensions at ambient temperature. The solid spheres were centrifuged, washed, and dried for further use. Then, the mesoporous silica shell was covered on solid silica spheres. Three hundred milligrams solid silica was dispersed in 50 mL deionized water by ultra-sonication for 45 min. And the silica suspensions were poured into a mixture of 60 mL ethanol, 450 mg CTAB, 90 mL water, and 1.7 mL NH_3_·H_2_O. After the mixture stirred for 60 min, TEOS (0.75 mL) was added. Subsequently, the nanoparticles were centrifuged after stirring for 6 h to collect samples and then re-dispersed in 40 mL water. About 1.2 g Na_2_CO_3_ was added into the water suspension with vigorous stirring. After the mixture was maintained at 55 °C for 12 h, the products of HMSS nanoparticles were collected and washed with anhydrous ethanol. The post-grafting method with the ratio of HMSS and APTES being at 4:1 (m/v) to prepare HMSS–NH_2_ at 80 °C under N_2_ condition for 8 h, to CTAB was removed by reflux [3].

#### Preparation of HMSS–N=N–COOH

The azobenzene-3,3′-dicarboxylic acid (50 mg) was added in pH 5.8 PBS. Then (5 mg/mL), EDC and (3 mg/mL) NHS were added to activate azobenzene-3,3′-dicarboxylic acid at 30 °C for 1 h. And 10 mL PBS containing 15 mg/mL HMSS–NH_2_ was added, and the mixture was stirred for 24 h. And the resultant HMSS–N=N–COOH was separated by centrifugation and washed with ethanol.

#### Preparation of HMSS–N=N–CS

0.15 g CS and 0.5 mL acetic acid were added in 50 mL water to prepare CS solution. And 100 mg HMSS–N=N–COOH was dispersed in 25 mL pH 5.0 PBS and activated by EDC and NHS for 0.5 h. Then, CS solution (10 mL) was poured in the suspension with continuous stirring for 1 day. Finally, the synthesized HMSS–N=N–CS was centrifuged and washed to collect the samples.

### Extraction of Colonic Enzyme Mixture from Microflora

The colonic microflora was collected according to a published work [37]. Then, the culture was inoculated to get enzyme mixture secreted by colonic microflora at 37 °C. The simulated colonic media containing enzyme mixture was filtered through a 0.22-μm filter to remove all the cellular debris from the culture fluid. Subsequently, the filtrate was lyophilized to obtain the enzyme mixture in the powder form, which was used in the further study.

### Drug Loading Process and Enzyme-Responsive Release

Twenty-five milligrams DOX was dissolved in 5 mL pH 3.5 HCl solution. And 100 mg HMSS–N=N–CS was added in the DOX solution and stirred at ambient temperature for 12 h. Subsequently, 0.2 M NaOH solution was used to adjust the pH of mixture to 7.0, and the suspension was stirred for another 12 h. Then, the DOX-loaded HMSS–N=N–CS (referred to HMSS–N=N–CS/DOX) was centrifuged and washed to remove the adsorbed DOX on the surface of HMSS–N=N–CS. The supernatant was gathered at each step to measure the DOX loading efficiency (LE) at 480 nm by UV-Vis spectrophotometry. The total mass of DOX loaded in HMSS–N=N–CS was calculated by subtracting the unloaded DOX after drug loading processes from the initial mass of DOX added. The HMSS/DOX was prepared as a control using HMSS as the initial carrier. The LE of DOX was calculated according to the equation:

$$ \mathrm{LE}\ \left(\%\right)=\frac{m_A-{m}_B}{m_A-{m}_B+{m}_C} \times 100 $$

In which *m*_A_ was the added mass of DOX, *m*_B_ was the mass of DOX in supernatant, and *m*_C_ was the total mass of HMSS–N=N–CS.

In vitro enzyme-responsive release of DOX from HMSS–N=N–CS/DOX was evaluated as follows. Two milligrams HMSS–N=N–CS/DOX and HMSS/DOX nanoparticles were dispersed in pH 7.4 PBS shaking at 125 rpm with different concentrations of colonic enzyme mixture (0 mg/mL, 0.3 mg/mL, and 1 mg/mL). At specified time intervals, 1 mL release medium was taken out to measure the absorbance. The release of DOX was measured at 480 nm. HMSS/DOX was used as a control.

### BSA Adsorption

The BSA adsorption amount was evaluated based on the published works [38, 39]. BSA was added in pH 7.4 PBS (0.5 mg/mL). Five milligrams HMSS and HMSS–NH_2_ and HMSS–N=N–CS were added into 2.5 mL PBS (pH 7.4). And the equal volume BSA solution was supplied, and the suspension was placed in a shaker at 100 rpm. After 6 h, centrifugation was used to collect the upper solution. At last, the BSA concentration was measured at 595 nm after being stained with Coomassie brilliant blue solution.

### Characterization

The mesoporous network structure and morphology of the HMSS nanoparticles were evaluated by TEM images (EM–208S, CSIS, USA). The surface area and pore size distribution of nanoparticles were characterized using nitrogen adsorption analysis analyzer (V-Sorb 2800P, Gold APP Instrument Corporation, China). The ξ potentials and particle sizes were characterized on a Nano-z90 Nanosizer (Malvern Instruments Ltd., Worcestershire, UK). TGA analysis was measured on a TGA-50 equipment (Shimadzu, Kyoto, Japan) with a heating rate of 10 °C/min under a nitrogen flow. Fourier transform infrared spectrophotometric (FT-IR) spectra were measured using a FT-IR spectrometer (Bruker Tensor27, Switzerland). The range was carried out from 400 to 4000 cm^−1^ using KBr pellet technique. Power XRD was performed on a Siemens D5005 X-ray diffractometer (Karlsruhe, Germany) with Cu-Kα radiation (*λ* = 1.5418 Å).

### Cell Culture and Cell Uptake Experiment

Caco-2 cells were cultured in a medium supplemented with 10% FBS, 1% nonessential amino-acid, 1% (v/v) pyruvic acid sodium, and 1% streptomycin. NIH-3T3 cells were cultured in DMEM with 1% streptomycin and 10% FBS. The Caco-2 cells uptake of the nanocarriers was characterized using FCM and CLSM. Caco-2 cells were seeded into 24-well plates. After culturing for overnight, free DOX, HMSS–N=N–CS/DOX, and HMSS–N=N–CS/DOX preincubated with colonic enzyme nanoparticles (equal to the concentration of 5 μg/mL DOX) were added to corresponding wells. After continued incubation for 2 h, the cell medium was removed and washed thoroughly with PBS. Then, the cells were fixed by 4% formaldehyde and stained by Hoechst 33258 for CLSM observation. FCM was used to obtain a quantitative evaluation of cellular uptake. Caco-2 cells were seeded in 6-well plates and further incubated for 24 h. After washing with PBS, the Caco-2 cells were incubated with free DOX, HMSS–N=N–CS/DOX, and HMSS–N=N–CS/DOX preincubated with colonic enzyme nanoparticles (equal to the concentration of 5 μg/mL DOX) in serum-free DMEM for 2 h. Then, the Caco-2 cells were rinsed with cold PBS, trypsinized, and re-suspended in 0.5 mL PBS. The DOX fluorescence in cells was measured using a FACS Canto flow cytometer (Becton, Dickinson, USA).

### In Vitro Cellular Proliferation Assay

The cytotoxicity of HMSS and HMSS–N=N–CS blank carriers towards NIH-3T3 and Caco–2 cells was testified by MTT assay [40, 41]. Briefly, Caco–2 cells and NIH-3T3 cells were separately seeded in 96-well plates and further incubated for overnight. The old cell medium was substituted by a serum–free medium containing different concentrations of nanoparticles. After incubation for 2 days, 50 μL of MTT solution (2 mg mL^–1^) was added and incubated for 4 h to measure the living cells. Then, MTT solution was removed, and 150 μL DMSO was added to dissolve formazan. Subsequently, the absorbance was measured on a microplate reader (Tecan, Männedorf, Switzerland) at 570 nm. The cytotoxicity of free DOX, HMSS–N=N–CS/DOX, and HMSS–N=N–CS/DOX preincubated with enzyme mixtures extracted from colonic microflora was measured using Caco-2 cells with the corresponding DOX concentrations of (0.1, 1, 5, 10, and 20 μg/mL). The incubation time was 48 h, and the other experiment processes were the same as above described.

### Toxicity Studies

The gastrointestinal mucosa irritation tests are vital for the evaluation of oral drug delivery in vivo biosafety. Male Sprague–Dawley rats (180 ± 10 g) were randomly divided into three groups (three rats for each group). Rats were administrated saline, HMSS, and HMSS–N=N–CS nanoparticles with a dose of 100 mg/kg for each day. After 7 days, all the rats were sacrificed, and the tissues were collected and examined by histopathological examination (H&E). To evaluate the biosecurity of HMSS and HMSS–N=N–CS nanoparticles, the body weights of BALB/c mice (18–20 g) were recorded after oral administration at a dose of 100 mg/kg for every other day. All experimental procedures were performed in accordance with the guidelines for the Care and Use of Laboratory Animals of Qiqihar Medical University and were approved by the Ethics Committee of Qiqihar Medical University.

### Statistics

Statistical data were analyzed with a SPSS software using two tail Student’s *t*-test. Error bars presented in this study are SD. A *p* < 0.05 is considered statistically significant.

## Results and Discussion

### Preparation and Characterization of HMSS–N=N–CS

**Fig. 1:**
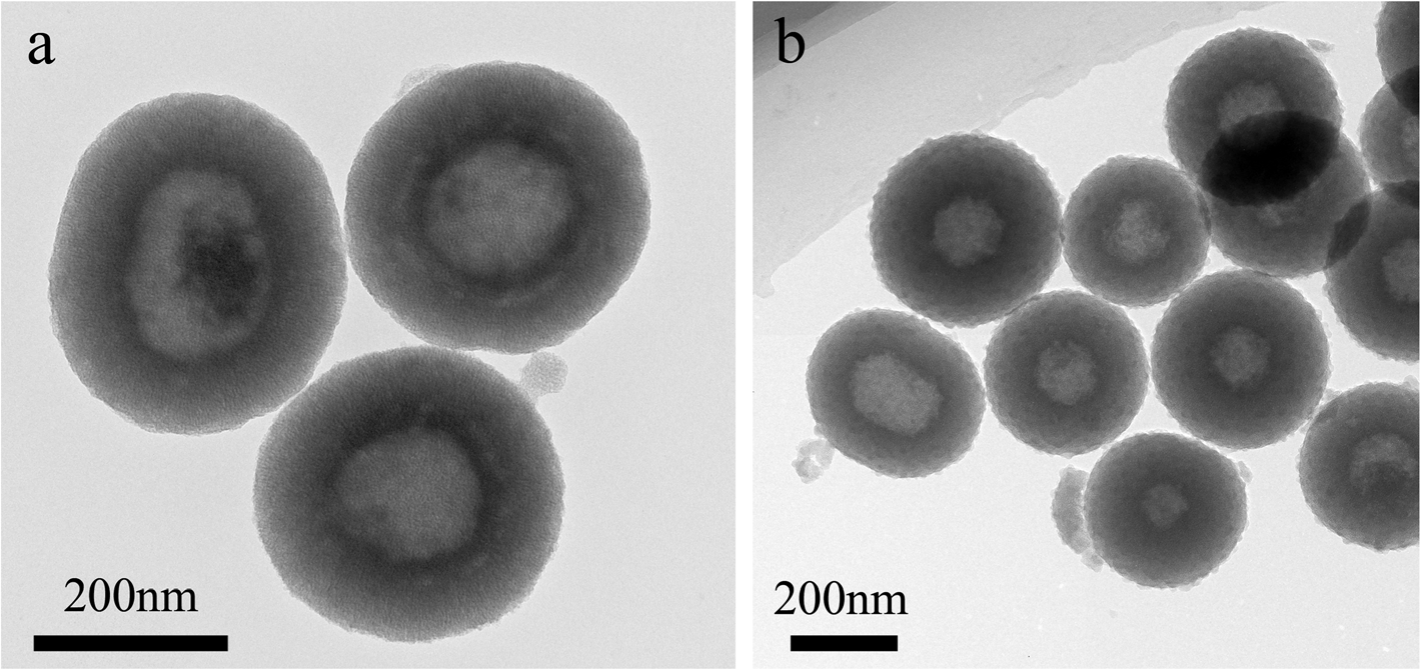
TEM of **a** HMSS and **b** HMSS–N=N–CS

HMSS were prepared based on previous works with minor changes [36]. First, solid SiO_2_ nanospheres were prepared, and a mesoporous shell was coated on the surface of the solid silica nanospheres containing the CTAB template. Then, Na_2_CO_3_ was used to selectively etch the solid SiO_2_ nanospheres while the mesoporous shell was protected by template CTAB. The preparation of HMSS–N=N–CS with CS attached as a “gatekeeper” by an azo linkage is described in Fig. 1a. First, the surfaces of HMSS nanoparticles were modified with APTES as an alkyl coupling reagent to become amino-functionalized HMSS (HMSS–NH_2_) by a postmodification method. Subsequently, HMSS–N=N–COOH was prepared by an amidation reaction between the amino groups of HMSS–NH_2_ and the carboxyl groups of azobenzene-3,3′-dicarboxylic acid. Then, CS was covalently modified onto the surface of HMSS nanoparticles by an amidation reaction between the carboxyl groups of HMSS–N=N–COOH and the amino groups in CS.

As displayed in the transmission electron microscopy (TEM) image in Fig. 1a, the average diameter of HMSS was 280 nm, and the HMSSs had a uniform hollow structure and highly ordered mesoporous shell. The average mesoporous shell thickness was approximately 90 nm. Compared to the smooth surface of HMSS, the surface of grafted polymer HMSS–N=N–CS (Fig. 1b) was rough, indicating that the CS covered the HMSS carrier.

**Fig. 2:**
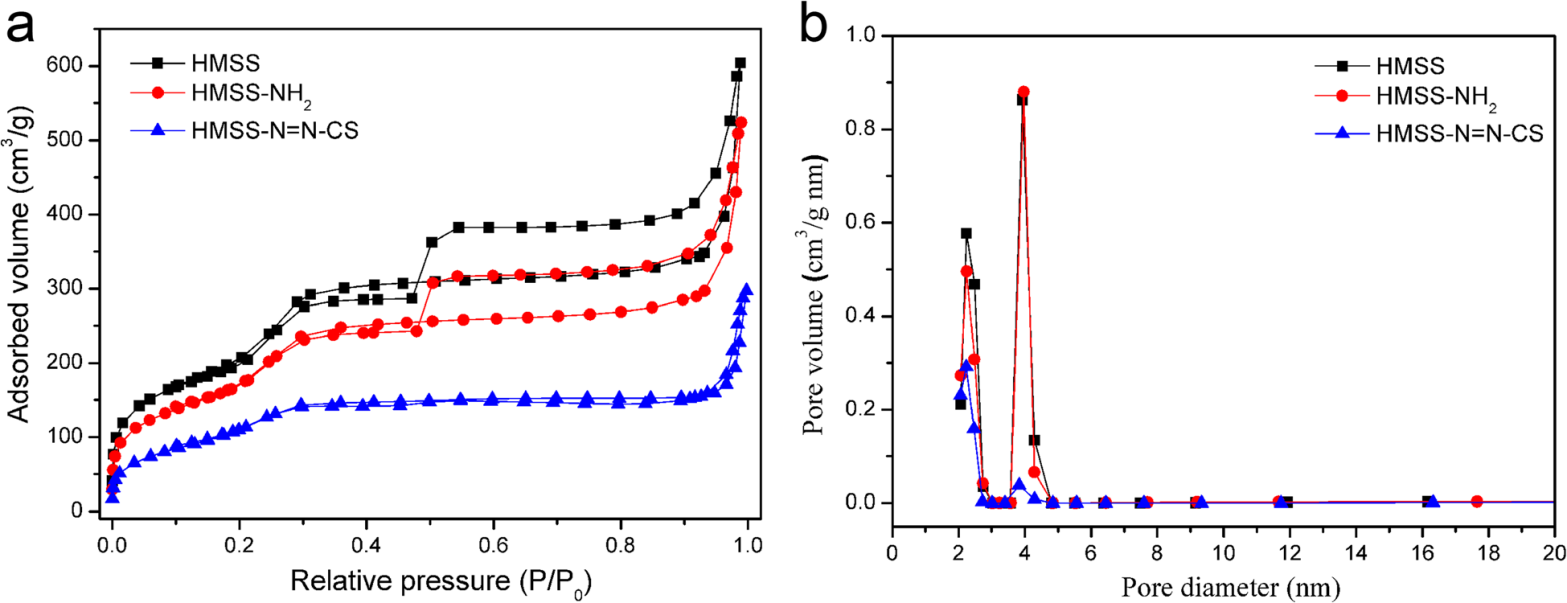
**a** The nitrogen adsorption/desorption isotherms and **b** pore size distributions of HMSS, HMSS–NH_2_, and HMSS–N=N–CS

The surface areas and pore distributions of mesoporous materials played a crucial role in loading and delivering host molecules for controlled release. Pore size distribution curves and isotherms were measured by N_2_ adsorption and desorption analysis (Fig. 2). Detailed parameters (the Brunauer–Emmett–Teller (BET) surface area (*S*_BET_), total pore volume (*V*_P_), and pore size distribution (*D*_P_)) are displayed in Table 1. The *S*_BET_ and *V*_P_ of pure HMSS were 810.7 m^2^/g and 0.969 cm^3^/g, respectively, and the *D*_P_ was approximately 3.8 nm. The *D*_P_ of HMSS–NH_2_ was almost the same as that of HMSS after amination, indicating that the mesopores were not blocked after amino functionalization. The *S*_BET_ and *V*_P_ of HMSS–N=N–CS were markedly decreased after modification by the azo compound and CS coating, indicating that CS had coated the surface of the HMSS [1].

**Table 1:** The *N*_2_ adsorption and desorption parameters of different functionalized HMSS nanoparticles

Sample	*S*_BET_ (m^2^/g)	*V*_P_ (cm^3^/g)	*D*_p_ (nm)
HMSS	810.7	0.969	3.8; 2.2
HMSS–NH_2_	688.1	0.822	3.8; 2.2
HMSS–N=N–CS	437.6	0.406	2.2; 3.8

**Fig. 3:**
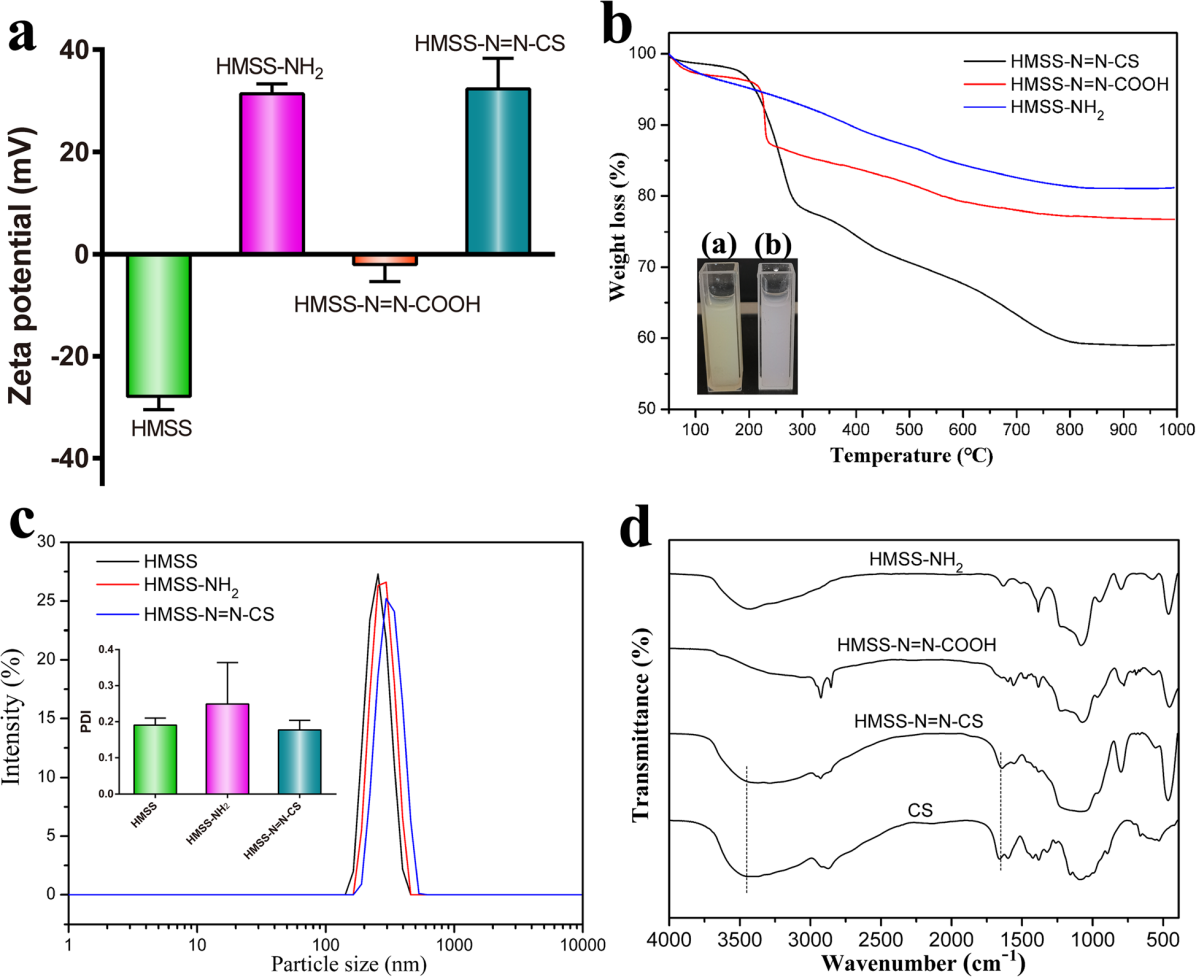
**a** The corresponding ξ potentials of HMSS, HMSS–NH_2_, HMSS–N=N–COOH, and HMSS–N=N–CS; **b** The TGA curves of HMSS–NH_2_, HMSS–N=N–COOH, and HMSS–N=N–CS (the inset: the photograph of (**a**) HMSS–N=N–COOH and (**b**) HMSS–NH_2_); **c** Size distribution of HMSS, HMSS–NH_2_, and HMSS–N=N–CS inset: the corresponding PDI values of nanoparticles; and **d** FT-IR spectra of HMSS–NH_2_, HMSS–N=N–COOH, HMSS–N=N–CS, and CS

**Fig. 4:**
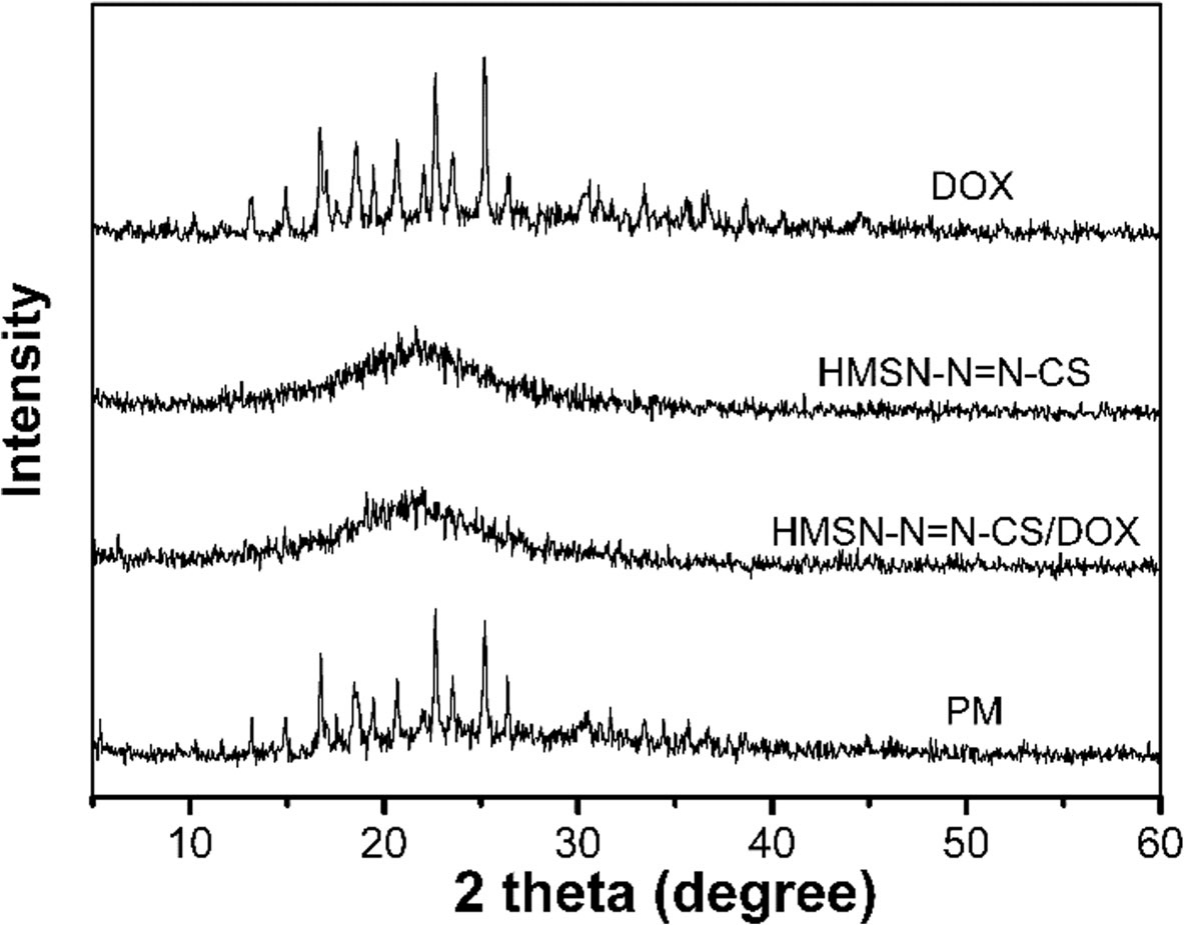
XRD patterns of DOX, HMSS–N=N–CS, HMSS–N=N–CS/DOX, and PM

The successful grafting of HMSS–N=N–CS was verified by various methods. The ξ potential of HMSS–NH_2_ underwent a great change after functionalization, varying from − 27.9 to + 31.4 mV, as shown in Fig. 3a, which was ascribed to the addition of the amine groups to the surface of HMSS. After HMSS–NH_2_ reacted with azobenzene-3,3′-dicarboxylic acid to form HMSS–N=N–COOH, the ξ potential further decreased to − 2.0 mV because of the carboxyl groups on the surface of the HMSS. After the CS polymer was further grafted onto the surface of the HMSS to form HMSS–N=N–CS, the ξ potential reverted to + 32.4 mV. The result was ascribed to the amino-abundant positively charged CS coating on the surface of the HMSS [1]. Thermogravimetric analysis (TGA) curves of HMSS, HMSS–NH_2_, HMSS–N=N–COOH, and HMSS–N=N–CS species are shown in Fig. 3b. Compared with HMSS–N=N–COOH, HMSS–N=N–CS lost an additional weight of approximately 19%, which was due to the removal of CS chains. The grafting of azo bonds on the surface of HMSS was also confirmed by a color change during the preparation of HMSS–N=N–COOH, as shown in the inset in Fig. 3b. The reactant HMSS–NH_2_ is white, while the product HMSS–N=N–COOH was yellowish-brown after the azobenzene-3,3′-dicarboxylic acid reacts with the amino groups of HMSS. The hydrodynamic diameter (*D*_H_) and polydispersity index (PDI) values of HMSS, HMSS–NH_2_, and HMSS–N=N–CS were determined in distilled water, as shown in Fig. 3c. The HMSS had a diameter of 309 nm and a PDI of 0.190. After the addition of amine groups on the surfaces of HMSS to form of HMSS–NH_2_, the *D*_H_ increased to 324 nm. The diameter of HMSS–N=N–CS was 342 nm, which was larger than that of HMSS–NH_2_ due to the grafted CS chains. The PDI of HMSS–N=N–CS (0.177) was smaller than that of HMSS–NH_2_, indicating that the average particle sizes had become even more after the grafting of CS. Compared with the diameters obtained from TEM, the diameters of HMSS and HMSS–N=N–CS measured by DLS were larger. The *D*_H_ of the nanoparticles was measured in a water environment with a hydration layer, while the size of the nanoparticles provided by TEM was obtained from dry nanoparticles [3]. FT-IR spectra of HMSS–NH_2_, HMSS–N=N–COOH, HMSS–N=N–CS, and CS are shown in Fig. 4d. Compared with the peaks for HMSN–NH_2_, an increase in the adsorption peaks at 2853 and 2925 cm^−1^ was attributed to the vibration of − CH_2_ in the grafting of carboxy-terminal azo bonds. After CS was added to the surface of HMSS–N=N–COOH, there were increases in the adsorption peaks at 1660 cm^−1^ and 3435 cm^−1^, which were attributed to υ_(C=O)_ in the amide band and the vibration of N–H in the CS. All the results proved the successful preparation of HMSS–N=N–CS.

### Drug State and Loading Efficiency

DOX was chosen to investigate the loading and release behaviors of HMSS–N=N–CS. When the pH value of the HMSS–N=N–CS nanoparticle suspension was adjusted to pH 3.5, the CS biopolymer became positively charged (the pK_a_ of CS was 6.3) due to the protonated amino groups in the acidic environment [24]. The CS polymer became positively charged and swelled, leading to the opening of mesopores of HMSS attributed to the repulsive interaction between CS charges. Thus, DOX gained access to the mesopores of HMSS–N=N–CS by diffusion. However, after the drug-loaded mixture was adjusted to 7.4, the CS chains deprotonated and collapsed to hinder the premature release of DOX.

The LE of HMSS–N=N–CS/DOX was 35.2%, which was much larger than that of other DOX-loaded mesoporous silica delivery systems [3, 16]. The high LE of DOX in HMSS nanocarriers was attributed to the hollow cavity, large surface area, and mesoporous network, which could be used as a drug reservoir. The physical state of DOX in HMSS–N=N–CS/DOX was evaluated by power X-ray diffraction (XRD). As shown in the XRD profiles (Fig. 4), raw DOX exhibited characteristic and intense drug crystalline diffraction peaks. The physical mixture (PM) of HMSS–N=N–CS and DOX also showed obvious crystalline diffraction peaks. However, no distinct crystalline peaks were exhibited by HMSS–N=N–CS/DOX, which proved that the physical state of DOX in HMSS–N=N–CS/DOX was noncrystalline because of the constraints of the mesoporous structure of HMSS.

### In Vitro Enzyme-Responsive Release in Simulated Colonic Environment

**Fig. 5:**
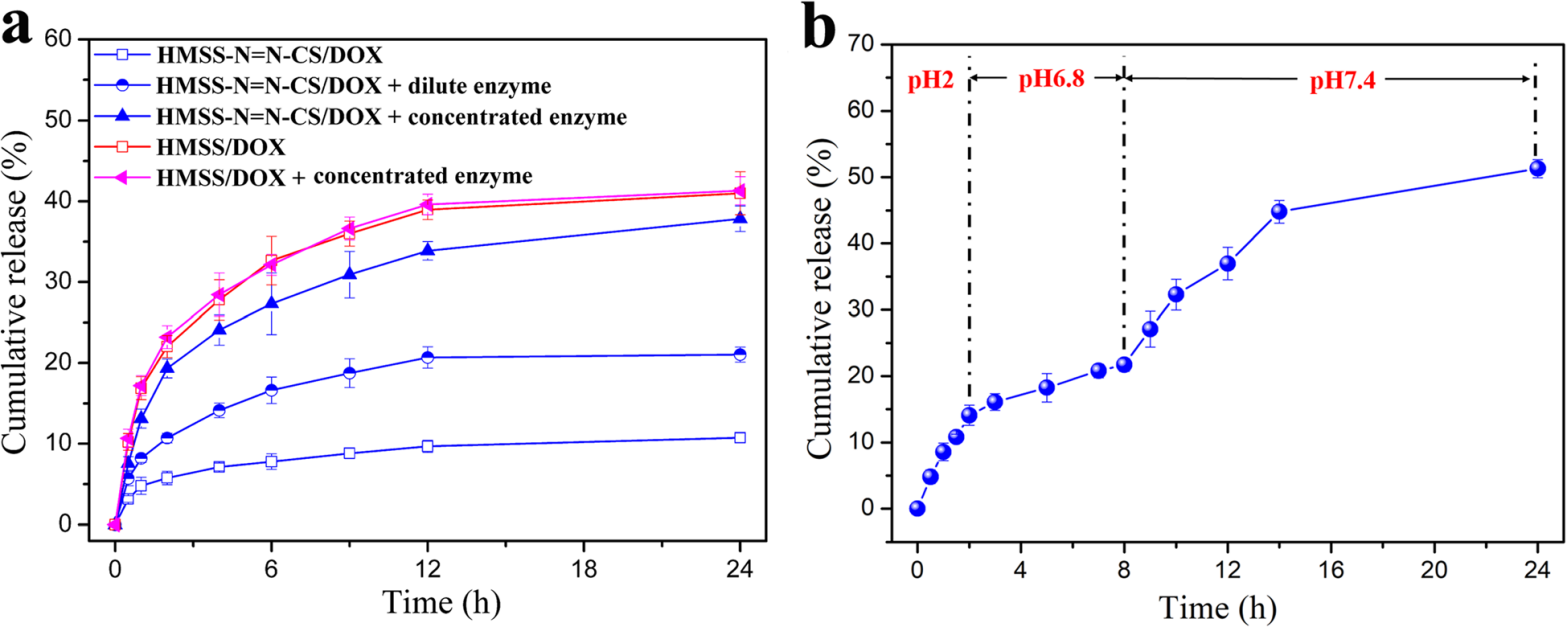
**a** Cumulative release profiles of HMSS/DOX and HMSS–N=N–CS/DOX in pH 7.4 PBS in presence of concentrated and dilute colonic enzyme mixture; and **b** in vitro pH-responsive release behaviors of DOX from HMSS–N=N–CS in the release media of different pH values

To investigate the enzyme-responsive release of HMSS–N=N–CS, HMSS–N=N–CS/DOX and HMSS/DOX nanoparticles were added to pH 7.4 PBS with different concentrations of colonic enzyme mixture. As displayed in Fig. 5a, HMSS–N=N–CS/DOX exhibited the slow release of DOX in PBS at pH 7.4, and the cumulative release percentage was only approximately 10% within 24 h, indicating a good capping capability of the CS polymers and azo bonds. As expected, in the case of dilute enzyme in PBS at pH 7.4, the cumulative release of DOX was improved to more than 20% within the same period. Additionally, the release amount of DOX was dramatically increased to nearly 40% in the presence of concentrated enzyme. Compared with the enzyme-responsive release from HMSS–N=N–CS/DOX, the release of DOX from HMSS/DOX had similar trends in the presence or absence of concentrated enzyme. The relatively low drug release percentage was due to the electrostatic interaction between the negatively charged HMSS and the positively charged DOX [42]. The above results proved that the release of DOX from HMSS–N=N–CS/DOX was markedly accelerated by enzymes extracted from microflora in colonic regions. The enzyme-responsive release mechanism could be that the azo bonds in HMSS–N=N–CS are degraded by the enzyme, causing the detachment of CS from the surface of HMSS and the fast release from HMSS. Azo bonds have been reported to be cleaved by enzymes secreted by colonic microflora [34, 35].

Additionally, to further evaluate the enzyme-responsive release from HMSS–N=N–CS/DOX in the mimetic GIT environment, the HMSS–N=N–CS/DOX nanoplatforms were initially dispersed in SGF for 2 h and then further dispersed in SIF for 6 h, and finally, the carriers were added to pH 7.4 PBS containing 1 mg/mL extracted enzyme. As shown in Fig. 5b, in simulated gastric juice, the release of DOX was relatively fast, and the cumulative amount reached 15% within 2 h. The relatively fast release was due to the weaker interaction between HMSS–N=N–CS and DOX under acidic conditions than under neutral conditions [1]. Then, the release of DOX was slowed down in SIF for 2–8 h. However, after the HMSS–N=N–CS/DOX was incubated with extracted enzymes in pH 7.4 PBS, the release of DOX continued to increase markedly, and the cumulative release amount reached more than 50% within 24 h. The incomplete DOX release from HMSS–N=N–CS/DOX was due to the strong interaction between the positively charged DOX and negatively charged HMSS.

### The Protein Adsorption by and Stability of HMSS–N=N–CS

**Fig. 6:**
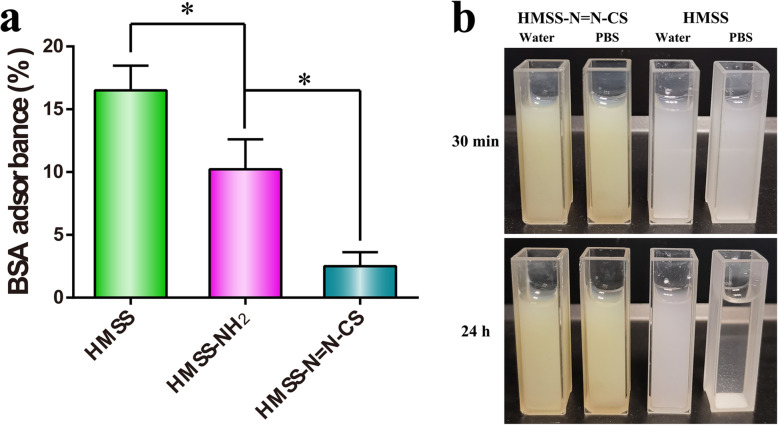
**a** BSA adsorbance amounts of HMSS and HMSS–NH_2_ and HMSS–N=N–CS (*n* = 3, *p < 0.05). **b** Photograph images of HMSS–N=N–CS and HMSS dispersed in water and PBS with a concentration of 4 mg/mL

For oral administration, the surface properties of nanocarriers will unavoidably affect drug release behaviors and bioadsorption [43]. An assay of protein adsorption on the surface was used to evaluate the effect of grafted CS on the surface of HMSS. As displayed in Fig. 6a, bare HMSS nanocarriers had a dramatic BSA adsorption of up to 16.5%, which was attributed to the large surface area and hollow cavity of HMSS, strong adsorption ability, and nonspecific interactions between silanol groups of HMSS and BSA [38, 39]. In addition, HMSS–NH_2_ similarly had a relatively high adsorbed BSA amount of 10.2%. Nevertheless, the percentage of adsorbed BSA on the surface markedly decreased to 2.5% after the polymer CS was added as a cover, thus dramatically decreasing the effect on the in vivo behavior of HMSS–N=N–CS. To further observe the stability of the HMSS–N=N–CS and HMSS samples, 20 mg of HMSS–N=N–CS and HMSS was added to pH 7.4 PBS and deionized water. As displayed in Fig. 6b, although HMSS–N=N–CS and HMSS were relatively stable in water, HMSS quickly flocculated in pH 7.4 PBS. By contrast, the dispersity of HMSS–N=N–CS was obviously enhanced after the polymer CS was grafted onto the surfaces of HMSS. Additionally, HMSS–N=N–CS carriers can remain stable for more than 12 h without precipitation in pH 7.4 PBS. These results proved that covering with the hydrophilic CS polymer could improve the dispersity and decrease protein adsorption on the surface of HMSS–N=N–CS.

### Cellular Uptake

**Fig. 7:**
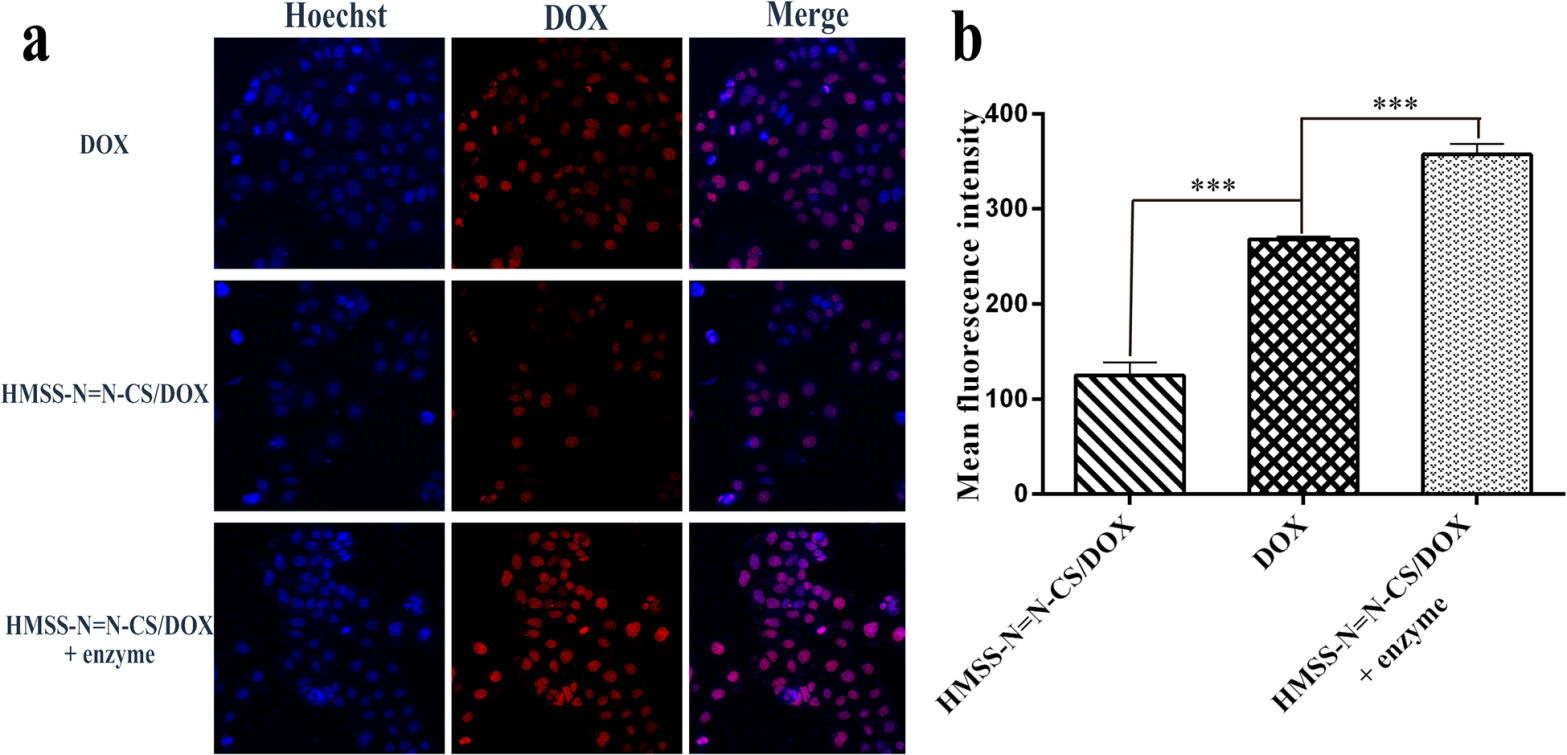
**a** CLSM of Caco-2 cells incubated with different samples. **b** The MFI of DOX, HMSS–N=N–CS/DOX, and HMSS–N=N–CS/DOX treated by enzyme measured by FCM in Caco-2 cells (*n* = 3, ***p < 0.001)

Caco–2 cells, as human epithelial colorectal adenocarcinoma cells, are widely used as model cells in oral drug delivery. As shown in Fig. 7a, Caco–2 cells incubated with free DOX showed a relatively strong fluorescence signal resulting from DOX because positively charged DOX could enter the cell and then the nucleus. Compared with the free DOX group, the HMSS–N=N–CS/DOX group showed a weaker fluorescence signal intensity due to incomplete drug release from HMSS–N=N–CS/DOX. However, after HMSS–N=N–CS/DOX was preincubated with the colonic enzyme mixture for 1 h, it showed a markedly increased fluorescence signal. This was attributed to the azo bonds being cleaved by the enzyme mixture, which led to the removal of the CS from the surfaces of HMSS, thus significantly accelerating the DOX release from HMSS–N=N–CS/DOX. To quantitatively evaluate the cellular uptake differences for HMSS–N=N–CS/DOX and HMSS–N=N–CS/DOX incubated with extracted enzymes, FCM was used. As shown in Fig. 7b, the mean fluorescence intensity (MFI) for the HMSS–N=N–CS/DOX group was 124.7, which was weaker than that of the free DOX group, with a *p* value less than 0.001. Excitingly, after HMSS–N=N–CS/DOX was preincubated with the colonic enzyme mixture for 1 h, the MFI markedly increased to 357 and even exceeded that of the free DOX group owing to the accelerated drug release from HMSS–N=N–CS/DOX after the breakage of azo bonds. All these results indicated that the azo bonds in HMSS–N=N–CS/DOX could be cleaved in the presence of colonic enzymes, which led to the shedding of CS from the surface of HMSS and accelerated the DOX release from HMSS.

### In Vitro Cell Viability Evaluation

**Fig. 8:**
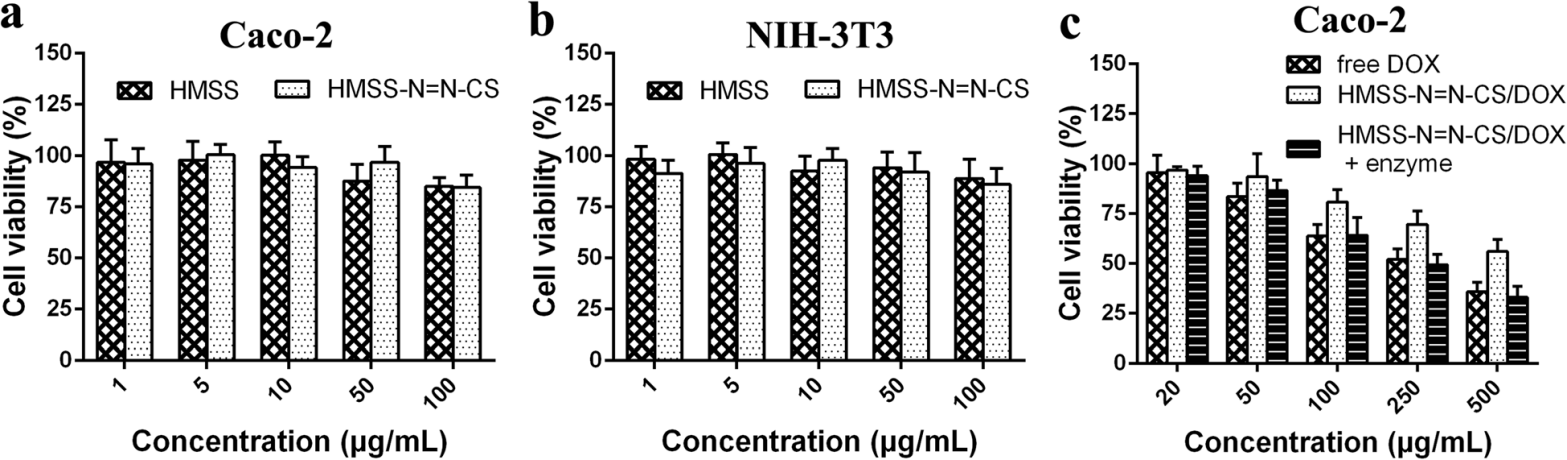
Effect of HMSS and HMSS–N=N–CS on cell proliferation of **a** Caco–2 cells and **b** NIH-3T3 cells for 48 h by MTT assay. **c** Cytotoxicity of free DOX, HMSS–N=N–CS/DOX, and HMSS–N=N–CS/DOX with enzyme against Caco-2 cells with different concentrations for 48 h

To prove the enzyme-responsive release effect of HMSS–N=N–CS/DOX in simulated colonic conditions, an in vitro cell viability assay was carried out using Caco-2 cells. The classic anticancer drug DOX was used in the cell viability assay. Prior to this assay, different concentrations of blank HMSS and HMSS–N=N–CS were employed to ascertain the biocompatibility of the nanoplatform towards Caco-2 cells and normal NIH-3T3 (mouse embryo fibroblast) cells at various concentrations from 10 to 250 μg/mL by MTT assay [44, 45]. As displayed in Fig. 8a, b, HMSS and HMSS–N=N–CS at different concentrations showed negligible cytotoxicity after incubation with Caco–2 cells or NIH-3T3 cells, and the viability of Caco–2 cells was 87.9% and 88.3% at the high concentration of 100 μg/mL, respectively, which is sufficiently high for clinical applications due to the high drug loading of HMSS. In addition, NIH-3T3 cells incubated with HMSS and HMSS–N=N–CS for 48 h had high cell viability (above 80%) at the relatively high concentration of 100 μg/mL. These results indicated that HMSS and HMSS–N=N–CS are cytocompatible and could be employed for oral delivery.

The effect of the DOX-loaded nanocarrier HMSS–N=N–CS/DOX on the viability of Caco–2 cells is displayed in Fig. 8c. Free DOX showed strong and concentration-dependent cytotoxicity towards 4T1 cells, which was attributed to the fact that positively charged free DOX could pass through 4T1 cell membranes easily. The IC_50_ value for the free DOX group was determined to be 10.18 μg/mL using the SPSS Statistics software. Compared with free DOX, HMSS–N=N–CS/DOX exhibited a higher cell viability at the same DOX concentration owing to the incomplete release of DOX from HMSS–N=N–CS induced by the strong electrostatic interactions between negatively changed HMSS carriers and positively changed DOX. The IC_50_ value for the HMSS–N=N–CS/DOX group was 32.22 μg/mL, which was much higher than that for free DOX. However, HMSS–N=N–CS/DOX preincubated with concentrated colon enzymes showed an obvious concentration-dependent cytotoxicity, and the IC_50_ value was calculated to be 9.41 μg/mL, which was much lower than that of HMSS–N=N–CS/DOX. This reason could be ascribed to the colon enzymes degrading the azo bonds in HMSS–N=N–CS, which would lead to the detachment of grafted CS from the surface of HMSS, causing the fast release of DOX from HMSS carriers.

### Toxicity Studies

**Fig. 9:**
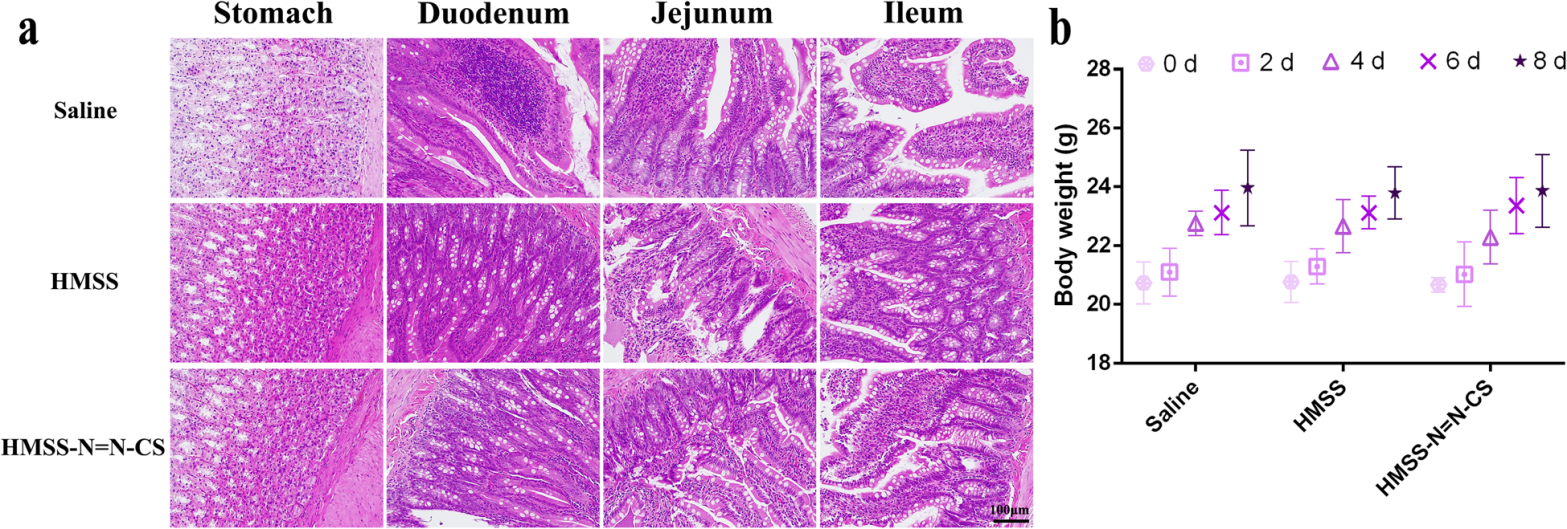
**a** Gastrointestinal mucosa irritation assay after oral administration of HMSS and HMSS–N=N–CS with the dose of 50 mg/kg for 7 days. **b** The weight changes of mice after oral administration for a week. Data were means ± SD (*n* = 3)

The hazards of using HMSS–N=N–CS are a vital factor to be considered before its clinical applications in the future. H&E staining of gastrointestinal mucosa irritation is essential to evaluate the in vivo biosafety of the delivery system for oral administration (Fig. 9a). Compared to a saline group, both the HMSS and HMSS–N=N–CS groups exhibited no marked histopathological changes or hyperemia after oral administration for a week with an administration dose of 100 mg/kg. No death or unusual behaviors of rats was observed during the experimental process. A decrease in body weight is widely regarded as an important and simple index for in vivo systemic toxicity [46]. As shown in Fig. 9b, the body weights of mice in the HMSS and HMSS–N=N–CS groups increased slightly and were similar to those of the saline group. The above results indicated that HMSS and HMSS–N=N–CS showed good biocompatibility as drug carriers for oral administration.

## Conclusions

In summary, biodegradable CS was attached through azo bonds to gate the openings of HMSS to achieve enzyme-responsive colon-specific drug delivery. DOX was loaded in the hollow cavity and mesopores of HMSS in a noncrystalline state with a high loading efficiency of 35.2%. Stability and BSA adsorption results illustrated that the CS gates could increase the biocompatibility and stability of HMSS. In vitro release results proved that HMSS–N=N–CS/DOX exhibited enzyme-responsive drug release behavior in the presence of colonic enzymes. CLSM uptake and FCM results indicated that the cellular uptake of DOX was obviously increased after HMSS–N=N–CS/DOX was incubated with the colonic enzyme mixture. Cell viability results indicated that HMSS–N=N–CS/DOX incubated with colonic enzymes showed increased cytotoxicity, and the IC_50_ value obviously decreased from 32.22 μg/mL for HMSS–N=N–CS/DOX to 9.41 μg/mL upon incubation.

## Data Availability

All data are fully available without restriction.

## Abbreviations

HMSS: Hollow mesoporous silica spheres
CS: Chitosan
DOX: Doxorubicin
DDSs: Drug delivery systems
MSSs: Mesoporous silica spheres
CLSM: Confocal laser scanning microscopy
FCM: Flow cytometry
FBS: Fetal bovine serum
LE: Loading efficiency
